# Comparison Between Standard Screw and Internal Brace in Treatment of Subtle Lisfranc Injury

**DOI:** 10.3390/jcm15031018

**Published:** 2026-01-27

**Authors:** Dong-Il Chun, Sanghoon Lee, Jaeho Cho, Sung Hyun Lee, Jeoung Wook Lee, Young Yi

**Affiliations:** 1 Department of Orthopedic Surgery, Soonchunhyang University Seoul Hospital, Seoul 04401, Republic of Korea; orthochun@gmail.com (D.-I.C.); blueye1014@naver.com (S.L.); 2 Department of Orthopedic Surgery, Chunchon Sacred Heart Hospital, Hallym University College of Medicine, Chuncheon 24253, Republic of Korea; chojaeho@hallym.or.kr; 3 Department of Orthopedic Surgery, CHA Bundang Medical Center, CHA University School of Medicine, Seongnam 13496, Republic of Korea; 4 Department of Orthopedic Surgery, Ewha Womans University Mokdong Hospital, Seoul 07985, Republic of Korea

**Keywords:** internal brace, Lisfranc injury, screw fixation

## Abstract

**Background:** Subtle Lisfranc injuries are low-energy, purely ligamentous lesions increasingly recognized in active patients; although screw fixation is common, Internal Brace (IB) flexible fixation is an alternative. **Methods:** In this multicenter retrospective study (2014–2021), 65 patients with subtle ligamentous Lisfranc injury (C1–M2 diastasis < 5 mm) underwent standard screw (SS, n = 35) or IB fixation (n = 30). Outcomes included AOFAS and VAS, standing radiographs and weight-bearing CT (WBCT) diastasis, pedobarography (4–6 months), and complications. **Results:** Demographics and injury mechanisms were similar. Both groups improved from preoperative status to final follow-up (*p* < 0.05). At 6 months, IB had higher AOFAS and lower VAS than SS (*p* < 0.05). Final stability was comparable: standing C1–M2 diastasis 2.54 mm (IB) vs. 2.55 mm (SS); WBCT dorsal 1.26 vs. 1.21 mm and plantar 3.58 vs. 3.42 mm (all NS). Pedobarography showed no significant side-to-side differences in either group. Complications favored IB: SS had screw breakage 11.4% (4/35), recurrent diastasis 2.9% (1/35), and early arthritis 5.7% (2/35); IB had no implant breakage, no severe recurrent diastasis, and no early arthritis. **Conclusions:** In this Level III study, IB fixation was associated with better 6-month clinical outcomes with similar final radiographic stability and fewer hardware-related complications versus SS.

## 1. Introduction

The Lisfranc joint, or tarsometatarsal (TMT) joint complex, is a keystone structure of the midfoot that maintains longitudinal and transverse arch stability during gait. Because the first through third rays function as a rigid lever for push-off, even small degrees of malalignment or residual instability at the TMT joints can translate inti substantial functional impairment. When Lisfranc injuries are missed or inadequately stabilized, patients may develop chronic midfoot pain, progressive collapse, decreased activity tolerance, and post-traumatic arthritis that can ultimately require salvage procedures such as arthrodesis [1,2]. Lisfranc injuries exist on a broad spectrum. High-energy fracture-dislocations are typically apparent on initial imaging and often mandate operative stabilization. In contrast, low-energy injuries—especially purely ligamentous patterns—may present with subtle symptoms and minimal displacement on standard radiographs [3]. These “subtle” Lisfranc injuries are increasingly encountered in younger, active individuals, including athletes, where symptoms may begin after a seemingly minor twist, axial load, or plantar flexion injury [4,5], a common mechanism in sports like football, gymnastics, and running [6,7]. The diagnosis can be challenging, as initial radiographs may appear normal in up to 50% of cases, leading to a high rate of missed or delayed diagnosis [8]. Accordingly, imaging evaluation has evolved from reliance on non-weight-bearing radiographs toward more functional and three-dimensional assessment. Weight-bearing radiographs can reveal diastasis or malalignment that is not evident without load, and comparative measures such as the distance between the medial cuneiform and second metatarsal base (C1–M2) are frequently used to quantify instability. When radiographs remain equivocal despite a strong clinical suspicion, advanced imaging is used to confirm ligament disruption and assess alignment. Computed tomography can define subtle joint incongruity and associated occult fracture, whereas magnetic resonance imaging can visualize Lisfranc ligament rupture and related soft-tissue injuries [9]. More recently, weight-bearing CT has been introduced to quantify midfoot alignment and diastasis under load with high spatial resolution, offering the potential for more reproducible measurement of subtle instability [10]. Despite these advances, there remains no universal consensus on a single diagnostic threshold, and clinical decision-making often incorporates symptom severity, physical examination, and imaging evidence of instability [4,8]. For consistency across centers, “subtle” injury in this study was defined as a purely ligamentous Lisfranc injury with C1–M2 diastasis < 5.0 mm on weight-bearing imaging.

The primary goal of treatment for Lisfranc injuries is to achieve and maintain an anatomic reduction of the TMT joint to restore a stable, plantigrade foot and minimize the risk of future degenerative changes [11]. Although subtle Lisfranc injuries may appear minimally displaced, treatment is guided primarily by stability rather than the absolute amount of diastasis. In general, stable injuries without radiographic or stress-provoked instability can be managed nonoperatively with immobilization and protected weight-bearing. For unstable ligamentous injuries, surgical stabilization is the consensus treatment. Trans-articular screw fixation has long been considered the gold standard, providing rigid fixation to allow for ligamentous healing [11]. However, this method is not without drawbacks. The inherent rigidity of screw fixation can impede normal joint micromotion, potentially leading to cartilage damage. Furthermore, screws are prone to breakage, and a second surgery for hardware removal is often required, which carries its own risks and delays the return to full activity.

Flexible fixation has evolved over “generations” in an effort to balance stability with physiologic motion. Suture-button constructs were introduced to provide dynamic stabilization; however, prior techniques may be limited by point fixation and potential elongation/creep over time, and may not fully address multiplanar or intercuneiform instability in certain patterns. The Internal Brace (IB) represents a newer augmentation strategy using a high-strength FiberTape-based construct intended to maintain reduction while permitting controlled micromotion, potentially reducing metal hardware failure and avoiding routine removal. Despite increasing adoption, clinical data specifically evaluating this third-generation IB technique in a clearly defined “subtle” purely ligamentous cohort—and direct comparisons with standard screw fixation—remain limited.

Importantly, the potential benefits of flexible fixation may be most relevant in the specific population of subtle, purely ligamentous Lisfranc injuries: these patients are often active, have relatively preserved joint surfaces, and may prioritize early functional recovery and avoidance of secondary surgery. At the same time, because the initial displacement is limited, the threshold for clinically significant loss of reduction is narrow and outcomes may be sensitive to small differences in fixation stability. Therefore, a focused comparison within a clearly defined subtle ligamentous cohort is needed to determine whether flexible fixation provides comparable radiographic stability to standard screw fixation while improving early clinical recovery and reducing hardware-related complications.

The purpose of this study was to compare clinical outcomes, radiographic maintenance of reduction, plantar pressure parameters, and complications between Internal Brace fixation and standard screw fixation in patients undergoing operative treatment for subtle, purely ligamentous Lisfranc injuries. We hypothesized that Internal Brace fixation would provide radiographic stability comparable to standard screw fixation, with improved early pain and functional outcomes and fewer hardware-related complications.

## 2. Materials and Methods

### 2.1. Study Design and Patient Selection

This study was a multicenter, retrospective comparative analysis of a serial cohort of patients treated for subtle Lisfranc injuries. Data was collected from four institutions: Inje University Seoul Paik Hospital, Chunchon Sacred Heart Hospital, Soonchunhyang University Seoul Hospital, and Sejong Sports Medicine & Performance Center. The study period was from March 2014 to March 2021. This study was conducted in accordance with the Declaration of Helsinki and was approved by the institutional review board of each participating center.

All consecutive patients who underwent operative treatment for a Lisfranc injury during the study period were screened (n = 348 feet) A “subtle Lisfranc injury” was defined as a: (1) purely ligamentous Lisfranc injury, isolated rupture of the Lisfranc ligament without an associated fracture fragment with (2) <5 mm diastasis between the medial cuneiform (C1) and the base of the second metatarsal (M2) on weight-bearing radiographs.

Inclusion criteria were: (1) diagnosis of purely ligamentous Lisfranc ligament rupture confirmed by clinical examination and imaging, (2) C1–M2 diastasis < 5 mm on initial weight-bearing radiographs.

Exclusion criteria were: (1) patients who received nonoperative treatment; (2) injuries involving extended tarsal ligaments; (3) high-energy trauma mechanisms; (4) patients with underlying neurologic impairment, generalized ligamentous laxity, or pathologic flatfoot; and (5) cases with insufficient or poor-quality radiographs for accurate measurement.

**Figure 1:**
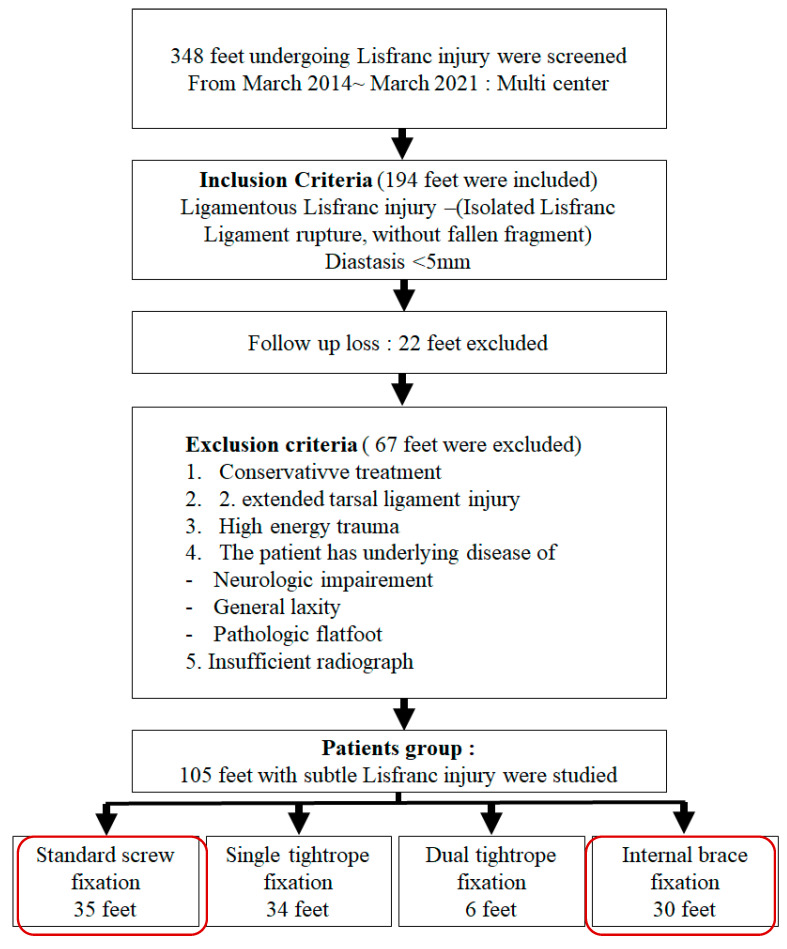
Flow chart of study group selection by means of the inclusion and exclusion criteria.

After applying these criteria, 194 feet were initially included. Twenty-two feet were lost to follow-up, and 67 feet were excluded based on the exclusion criteria. The final study cohort consisted of 105 feet in 105 patients with a subtle Lisfranc injury. These patients were then divided into four groups based on the surgical fixation method used: standard screw fixation (35 feet), single tightrope fixation (34 feet), dual tightrope fixation (6 feet), and Internal Brace fixation (30 feet). For the purpose of this paper, we focused on the direct comparison between the standard screw fixation and the Internal Brace groups (a total of 65 feet in 65 patients; SS n = 35 and IB n = 30) (Figure 1).

The diagnosis of a subtle Lisfranc injury was confirmed through a combination of clinical examination and radiological imaging. Radiographic evaluation included bilateral weight-bearing anteroposterior (AP) and lateral foot radiographs. The primary diagnostic criterion was the side-to-side difference in the diastasis between the medial cuneiform (C1) and the base of the second metatarsal (M2). A weight-bearing CT scan was utilized in many cases to provide a more detailed assessment of both dorsal and plantar diastasis [12]. In cases where the diagnosis remained equivocal, an MRI was performed to directly visualize and confirm the rupture of the Lisfranc ligament.

### 2.2. Surgical Procedure and Postoperative Protocol

All procedures were performed by fellowship-trained foot and ankle surgeons using a standardized dorsal approach, with the patient in the supine position under tourniquet control. In all cases, the goal was an anatomic reduction of the Lisfranc complex and restoration of normal alignment at the first and second TMT joints.

Reduction technique (both groups): After a dorsal incision between the first and second metatarsals, the interval was developed to expose the Lisfranc articulation. The joint was cleared of interposed tissue as needed. Reduction was obtained under direct visualization and fluoroscopy, typically using a pointed reduction clamp to approximate the medial cuneiform (C1) and the second metatarsal base (M2). Temporary fixation with a Kirschner wire was used at the surgeon’s discretion to maintain reduction before definitive fixation. Adequacy of reduction was confirmed on fluoroscopic AP, lateral, and 30° oblique views, ensuring restoration of C1–M2 alignment and absence of dorsal/plantar subluxation.

Standard Screw Fixation (SS): Following reduction, a 3.5-mm or 4.0 mm fully threaded cortical screw was placed from the medial cuneiform into the base of the second metatarsal in a lag fashion, under fluoroscopic guidance, taking care to avoid intra-articular penetration.

**Figure 2:**
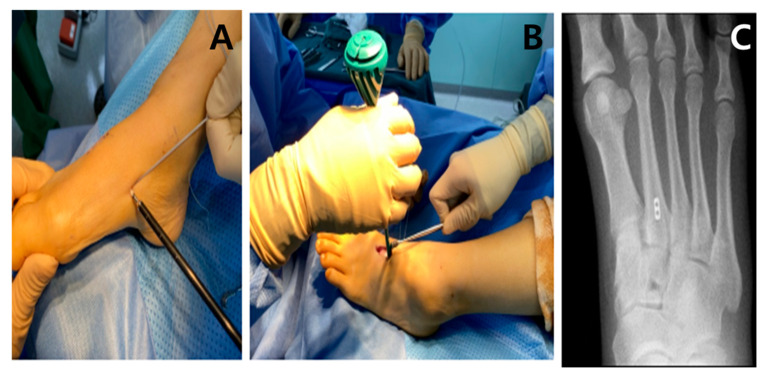
Surgical techniques for stabilization. (**A**,**B**) Internal Brace technique with suture tape and anchors providing flexible fixation across the joint. (**C**) Postoperative weight-bearing radiograph demonstrating that swiveLock anchor secures the FiberTape under tension, providing knotless fixation.

Internal Brace Fixation (IB): After reduction, Internal Brace stabilization was performed using two knotless suture anchors (SwiveLock; Arthrex, Naples, FL, USA) connected with FiberTape (Arthrex). A guidewire and drill were used to prepare anchor sites according to the manufacturer’s technique guide. One anchor was placed in the base of the second metatarsal and the second anchor in the medial cuneiform, creating a C1–M2 suture-tape construct. The FiberTape was tensioned while maintaining reduction under fluoroscopy, and the second anchor was secured to provide stable fixation while allowing controlled micromotion. Final fluoroscopic images confirmed maintained reduction, appropriate anchor position, and absence of joint violation (Figure 2).

The postoperative protocol was standardized across both groups. Patients were kept non-weight-bearing in a splint for 4 weeks. This was followed by partial weight-bearing in a walking boot from 4 to 6 weeks, during which active ankle range-of-motion exercises were initiated. Full weight-bearing with an arch-supporting insole was allowed at 6 weeks and continued until 12 weeks postoperatively. A gradual transition to regular footwear and activities was permitted after 12 weeks, with an anticipated return to sports practice between 12 and 16 weeks. For patients in the screw fixation group, hardware removal was typically performed between 4 and 6 months post-surgery, depending on clinical and radiographic signs of healing.

### 2.3. Outcome Assessment

Patients were followed up at serial intervals of 1, 6, and 12 months, and annually thereafter. The minimal follow-up duration for inclusion was 6 months. The average follow-up was 13.2 months (range, 6 to 26 months) for the standard screw group and 7.4 months (range, 6 to 26 months) for the Internal Brace group.

Clinical Evaluation: Clinical outcomes were measured preoperatively and at each follow-up visit using the Visual Analog Scale (VAS) for pain and the American Orthopaedic Foot & Ankle Society (AOFAS) midfoot scale.

Radiological Evaluation: Radiographic measurements were performed preoperatively, immediately postoperatively, and at the final follow-up. Measurements were taken from weight-bearing AP foot radiographs (C1–M2 diastasis, side-to-side difference) and weight-bearing CT scans (dorsal and plantar diastasis).

**Figure 3:**
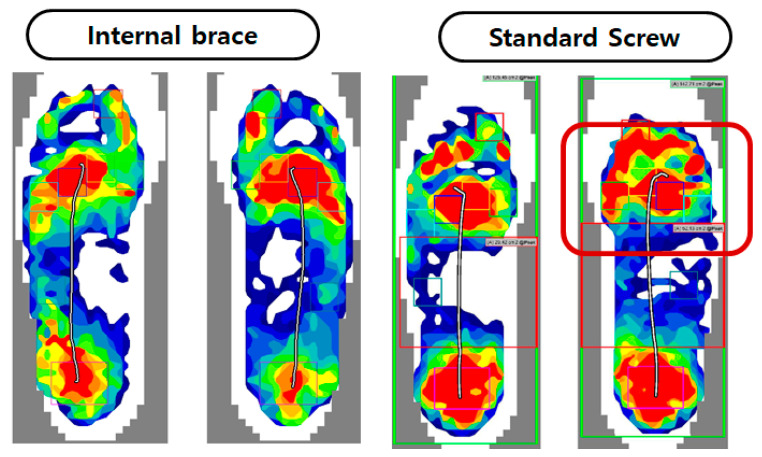
Comparison of foot pressure using pedobarograph with standard screw and internalbrace. Metatarsal foot pressure shows no different compare with contralateral foot in both group.

Plantar Pressure Measurement: Plantar foot pressure analysis was conducted between 4 and 6 months postoperatively using a pedobarography system (Figure 3) (prior to hardware removal in the screw group) to assess dynamic foot function and weight distribution. Patients walked barefoot at a self-selected speed across the platform; three valid trials were recorded per foot, and peak pressure and force-time integral were calculated for predefined foot regions.

Complications: Any complications, including infection, nerve injury, hardware failure (breakage or loosening), loss of reduction (recurrent diastasis), and development of post-traumatic arthritis, were recorded.

### 2.4. Statistical Analysis

Statistical analysis was performed using SPSS software (Version 25.0, IBM Corp., Armonk, NY, USA). An independent *t*-test was used to compare continuous variables between the two groups. Repeated measures ANOVA with a post hoc test was used to analyze changes in clinical and radiological parameters over time within each group. A *p*-value of less than 0.05 was considered statistically significant.

## 3. Results

### 3.1. Patient Characteristics

The standard screw (SS) group consisted of 35 patients with an age range of 18 to 69 years. The Internal Brace (IB) group included 30 patients aged 20 to 63 years. There were no significant differences in age, sex, or mechanism of injury between the two groups. (Table 1).

### 3.2. Clinical and Radiological Outcomes

**Figure 4:**
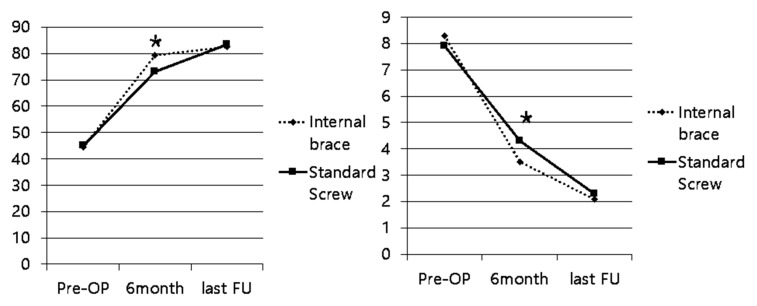
Clinical Outcome Scores. Data are presented as mean ± standard deviation. * Indicates a statistically significant difference (*p* < 0.05) between the Internal Brace and Standard Screw groups at the 6-month time point.

Both groups showed significant improvement in AOFAS and VAS scores from pre-surgery to the final follow-up (*p* < 0.05). At the 6-month follow-up, the IB group demonstrated significantly higher AOFAS scores and significantly lower VAS scores compared to the SS group (*p* < 0.05 for both) (Figure 4).

Radiologically, both fixation methods achieved and maintained a satisfactory reduction of the Lisfranc joint. Preoperatively, there was no significant difference in the amount of diastasis between the groups on either standing radiographs or weight-bearing CT scans. Postoperatively and at the final follow-up, both groups showed a significant reduction in diastasis compared to the preoperative measurements (*p* < 0.05). There were no statistically significant differences in the final C1–M2 diastasis, side-to-side difference, or dorsal/plantar diastasis on CT between the SS and IB groups (Table 2).

Plantar Pressure Analysis performed at 4 to 6 months after surgery showed no significant differences in peak pressure or force-time integral under the metatarsal heads when compared to the contralateral, uninjured foot in either group. This indicated a restoration of relatively normal weight distribution during gait for both fixation methods at this time point (Figure 3).

### 3.3. Complications

In the standard screw group (n = 35), there were four cases of screw breakage (11.4%) identified on follow-up radiographs before the planned removal. One of these cases was associated with a recurrent diastasis (2.9%). Additionally, two patients (5.7%) in the screw group developed radiographic evidence of early post-traumatic arthritis at the TMT joints.

**Figure 5:**
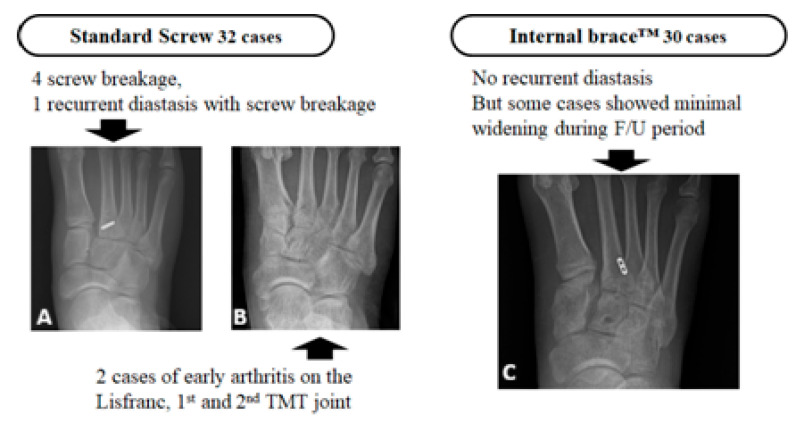
Complications in the standard screw fixation group. (**A**) Radiograph showing a broken Lisfranc screw. (**B**) Radiograph of a different patient showing early arthritic changes with joint space narrowing at the first and second TMT joints. (**C**) Radiograph from the Internal brace^TM^ group showing maintained reduction without recurrent diastasis, with only minimal interval widening during the follow-up period.

In the Internal Brace group (n = 30), there were no instances of implant breakage or severe recurrent diastasis requiring revision surgery. However, in some cases, a minimal widening of the C1–M2 interval was observed radiologically at the final follow-up compared to the immediate postoperative films, though this was not statistically significant and did not correlate with worse clinical outcomes. No cases of early arthritis were noted in the IB group at the final follow-up (Figure 5).

## 4. Discussion

The main finding of this study is that patients treated with Internal Brace (IB) fixation demonstrated significantly better early clinical outcomes at 6 months—higher AOFAS midfoot scores and lower VAS pain scores—than those treated with standard screw (SS) fixation, while maintaining comparable radiographic stability at final follow-up. These results suggest that dynamic fixation with IB may facilitate early functional recovery without compromising maintenance of reduction in subtle, purely ligamentous Lisfranc injuries.

The primary finding of our study was the significantly better clinical outcomes observed in the Internal Brace group at the 6-month follow-up. Patients treated with the Internal Brace reported less pain and had higher functional scores compared to those treated with screw fixation. This difference is likely multifactorial. First, the Internal Brace is a form of dynamic, rather than static, fixation. It is designed to allow for physiologic micromotion at the TMT joint, which may be less irritating to the articular cartilage and surrounding soft tissues than a rigid trans-articular screw. This concept is similar to the evolution of treatment for syndesmotic injuries, where flexible fixation has been shown to result in better functional outcomes [13]. Second, the improved early outcomes may be related to the avoidance of a second surgery. Patients in the screw fixation group were aware that another procedure would be required for hardware removal, which can be a source of apprehension and may lead them to subconsciously limit their activity levels. The Internal Brace, in contrast, typically does not require removal, allowing for a more streamlined and uninterrupted recovery process. This is a considerable advantage, as it eliminates the risks, costs, and recovery time associated with a second operation.

From a radiological standpoint, both techniques proved effective in reducing the Lisfranc diastasis and maintaining that reduction through the follow-up period. There were no significant differences in the final diastasis measurements between the two groups, indicating that the flexible Internal Brace provides stability comparable to that of a rigid screw for these low-energy injuries. This is a critical finding, as it validates the biomechanical principle of dynamic stabilization for this specific injury pattern. Our results are consistent with biomechanical studies that have shown suture-button constructs to have comparable or superior resistance to diastasis compared to screw fixation under cyclic loading conditions [14,15].

The complication rates in our study highlight a key differentiator between the two techniques. The 11.4% rate of screw breakage in our cohort is consistent with rates reported in the literature, which range from 10% to 30% [14]. While not all broken screws are symptomatic or lead to a loss of reduction, they represent a technical failure of the implant and can make subsequent removal more challenging. The single case of recurrent diastasis in the screw group occurred in a patient with hardware breakage, underscoring the potential for rigid fixation to fail under physiologic loads. In contrast, the Internal Brace group experienced no implant failures. This inherent material advantage of a high-strength, flexible suture over a rigid metal screw is a significant factor in favor of dynamic fixation.

Furthermore, the development of early arthritic changes in two patients in the screw group is concerning. Although our follow-up is relatively short, this finding may support the hypothesis that rigid trans-articular fixation can lead to increased chondral pressure and subsequent cartilage wear. By permitting more natural joint movement, the Internal Brace may be chondroprotective and potentially reduce the long-term incidence of post-traumatic osteoarthritis, a devastating complication of Lisfranc injuries. However, longer-term follow-up studies are needed to definitively confirm this potential benefit.

**Figure 6:**
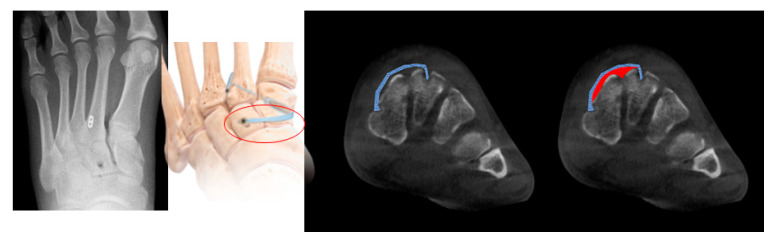
Intercuneiform fixation causes the fiber-wire to travel to the extraosseous area, which can change the tension due to the decrease in soft tissue.

We did observe a tendency for minimal, non-significant widening of the C1–M2 interval over time in the Internal Brace group. This phenomenon has been described with other flexible fixation devices and may be attributed to several factors. One possibility is a “settling” of the implant as the soft tissues heal and remodel. Another potential cause, as noted in our discussion slides, is related to the surgical technique in cases with associated intercuneiform instability. If the FiberTape is passed in an extraosseous plane to address intercuneiform diastasis, subsequent reduction of soft tissue swelling could lead to a minor loss of tension on the tape. This highlights the importance of meticulous surgical technique and suggests that in cases of combined Lisfranc and intercuneiform instability, fixation strategies may need to be adjusted (Figure 6).

Despite this subtle radiological finding, it is important to emphasize that it did not correlate with any recurrent clinical instability or inferior patient outcomes in our cohort.

### 4.1. Limitations

This study has several limitations. First, its retrospective nature introduces the potential for selection bias. Although we had standardized inclusion criteria, the choice of implant was ultimately at the discretion of the treating surgeon. It is possible that patients with slightly more pronounced instability or intercuneiform diastasis were preferentially treated with the Internal Brace, which could bias the results. We attempted to control for this by limiting the study to injuries with less than 5 mm of diastasis. Second, the follow-up period was relatively short. This limits our ability to draw definitive conclusions about the long-term prevention of osteoarthritis. Future studies with longer-term follow-up are essential. Third, we did not directly compare the Internal Brace to other forms of flexible fixation, such as the single or dual TightRope techniques [16]. Although our initial cohort included these patients, the numbers were too small for a robust statistical comparison [17]. Fourth, we observed a tendency toward minimal, non-significant widening of the C1–M2 interval over time in some Internal Brace cases. Because this cohort included patients with potentially complex or combined instability patterns (e.g., concomitant intercuneiform instability), we cannot fully determine whether this minor loss of reduction reflects an inherent limitation of the flexible construct (e.g., gradual suture-tape elongation/settling) or is primarily related to surgical technique factors such as fixation configuration, tunnel/anchor placement, or tensioning in complex instabilities. Finally, while we collected plantar pressure data, assessment was limited by the retrospective design; raw plantar-pressure datasets were not consistently archived across centers, precluding comprehensive quantitative and statistical comparisons between injured versus contralateral feet and between treatment groups. Also, single dynamic measurement may not fully capture the complex biomechanical changes and functional limitations experienced by patients during a wider range of activities.

### 4.2. Practical Applications

For patients with subtle, purely ligamentous Lisfranc injuries requiring operative stabilization, IB fixation may be considered a practical alternative to transarticular screw fixation when early functional recovery and avoidance of routine implant removal are prioritized. Our data suggest that IB can maintain reduction comparable to SS while reducing hardware-related events such as screw breakage. Surgeons should be aware that minimal interval widening may occur with flexible constructs and should ensure meticulous technique, particularly when concomitant intercuneiform instability is present. In clinical practice, postoperative assessment using weight-bearing imaging may help confirm maintenance of alignment during the early rehabilitation period.

In conclusion, the results of this comparative study demonstrate that for the treatment of subtle, purely ligamentous Lisfranc injuries, the Internal Brace technique provides comparable radiological stability to the traditional standard screw fixation. However, the Internal Brace is associated with significantly better early clinical outcomes, a lower rate of hardware-related complications, and the major advantage of obviating the need for a second surgery for hardware removal. These benefits make the Internal Brace an attractive and reproducible treatment option, particularly for young, athletic individuals who wish to return to high-level activities as quickly and safely as possible. Further prospective, randomized trials with long-term follow-up are warranted to confirm these findings and to further establish the role of dynamic fixation in the management of these increasingly common sports-related injuries.

## 5. Conclusions

In this multicenter retrospective cohort study of subtle, purely ligamentous Lisfranc injuries (C1–M2 diastasis < 5 mm on weight-bearing radiographs), Internal Brace fixation provided radiographic maintenance of reduction comparable to standard transarticular screw fixation, while demonstrating better early clinical outcomes (improved pain and function at 6 months) and fewer hardware-related complications, particularly avoiding screw breakage and the routine need for implant removal. These findings support Internal Brace fixation as a motion-preserving, less hardware-dependent alternative for carefully selected patients with subtle ligamentous Lisfranc instability.

Several limitations should be acknowledged. First, the retrospective, nonrandomized design introduces potential selection and surgeon-preference bias. Second, follow-up duration was relatively limited—particularly for the Internal Brace group—restricting conclusions regarding long-term durability and post-traumatic arthritis. Third, although key measurements were standardized (weight-bearing radiographs/CT-based diastasis), variability across centers in imaging availability and assessment may affect uniformity. Finally, the results apply specifically to subtle ligamentous injuries and may not generalize to fracture-dislocations or more severe Lisfranc patterns.

Future research should include prospective comparative studies (ideally randomized) with standardized imaging protocols, broader patient-reported outcome measures, and longer-term follow-up to determine whether dynamic fixation influences return to sport, durability of reduction, and the incidence of post-traumatic arthritis. Additional work evaluating cost-effectiveness and defining the optimal indications (including associated intercuneiform instability) will further clarify the clinical role of Internal Brace fixation in Lisfranc injury management.

## Figures and Tables

**Table 1:** Patient characteristics.

Characteristic	Standard Screw (SS) (n = 35)	Internal Brace (IB) (n = 30)
Age, years (mean ± SD)	38.6 ± 12.4	36.9 ± 11.8
Age, years (range)	18–69	20–63
Sex, male/female	24/11	20/10

Data are presented as mean ± SD, range, or n.

**Table 2:** Radiological Outcome Measures (Diastasis in mm).

Parameter	Group	Pre	Post	Last FU
Standing X-ray				
Diastasis	Internal brace	4.61 *	2.50	2.54
	Standard Screw	4.56 *	2.52	2.55
Side to side difference	Internal brace	2.68 *	2.56	2.62
	Standard Screw	2.65 *	2.55	2.58
Weight bearing CT				
Diastasis dorsal	Internal brace	3.58 *	1.18	1.26
	Standard Screw	3.52 *	1.21	1.21
Diastasis plantar	Internal brace	5.74 *	3.45	3.58
	Standard Screw	5.61 *	3.41	3.42

* Data are presented as mean values. No statistically significant differences were found between the groups at the final follow-up for any radiological parameter.

## Data Availability

The original contributions presented in this study are included in the article. Further inquiries can be directed to the corresponding author(s).
